# Investigating immune profile by CyTOF in individuals with long-standing type 1 diabetes

**DOI:** 10.1038/s41598-023-35300-7

**Published:** 2023-05-20

**Authors:** Helen Larsson, Sofie Albinsson Högberg, Marcus Lind, Hardis Rabe, Christine Lingblom

**Affiliations:** 1 Department of ENT, Head and Neck Surgery, NU Hospital Group, Trollhättan, Sweden; 2 Department of Otorhinolaryngology, Head and Neck Surgery, Institute of Clinical Sciences, Sahlgrenska Academy, University of Gothenburg, Göteborg, Sweden; 3 Department of Infectious Diseases, Institute of Biomedicine, The Sahlgrenska Academy, University of Gothenburg, Guldhedsgatan 10A, 41346 Göteborg, Sweden; 4 Department of Medicine, NU Hospital Group, Uddevalla, Trollhättan, Sweden; 5 Department of Molecular and Clinical Medicine, Institute of Medicine, The Sahlgrenska Academy, University of Gothenburg, Göteborg, Sweden; 6 Department of Medicine, Sahlgrenska University Hospital, Göteborg, Region Västra Götaland Sweden; 7 RISE Research Institutes of Sweden, Bioscience and Materials, Göteborg, Sweden; 8 Department of Clinical Microbiology, Sahlgrenska University Hospital, Göteborg, Region Västra Götaland Sweden

**Keywords:** Immunology, Biomarkers, Endocrinology

## Abstract

Type 1 diabetes (T1D) is an autoimmune disease caused by T-cell mediated destruction of pancreatic beta cells. Eosinophils are found in pancreatic tissue from individuals with T1D. Eosinophilic suppression of T cells is dependent of the protein galectin-10. Little is known when it comes to the role of eosinophil granulocytes in type 1 diabetes. Here we show that individuals with long-standing T1D had lower levels of galectin-10^hi^ eosinophils and a subgroup of galectin-10^hi^ eosinophils were entirely absent in all T1D patients. In addition, 7% immature eosinophils were present in the circulation of T1D patients whereas 0.8% in healthy individuals. Furthermore, higher levels of CD4+CD8+ T cells and Th17 cells were observed in patients with T1D. Blood samples from 12 adult individuals with long-standing T1D and 12 healthy individuals were compared using cytometry by time-of-flight. Lower levels of galectin-10^hi^ eosinophils, which are potent T cell suppressors, in individuals with T1D could indicate that activated T cells are enabled to unrestrictedly kill the insulin producing beta cells. This is the first study showing absence of galectin-10^hi^ eosinophilic subgroup in individuals with T1D compared with healthy controls. This study is a first important step toward unraveling the role of the eosinophils in patients with T1D.

## Introduction

There is a huge gap of knowledge when it comes to the role of eosinophil granulocytes in type 1 diabetes (T1D). T1D is an autoimmune disease caused by T-cell mediated destruction of pancreatic beta cells^1^. In rats, eosinophils migrate towards the Langerhans islets just before onset of T1D^2^ and the eosinophil-recruiting chemokine, eotaxin, and the high-affinity IgE receptor (FcεRI) are up-regulated in the pancreatic lymph nodes^3^. However, it was never clarified why the eosinophils are recruited to the pancreas. Recently, a report showed that CD49d+ eosinophils were reduced in type 1 diabetic mice vs control mice, and they could rule out that the reduced expression was not affected by high glucose^4^, indicating that eosinophils are impaired from the onset of T1D. In addition, eosinophils have been found in pancreatic tissue donated from patients with T1D^5^. Whilst it is unknown what the triggering factors causing diabetes are, it is known that autoreactive effector T cells kill the beta cells that produce insulin in the islets of Langerhans^6^. In the last decade, several reports have demonstrated that eosinophils have immunoregulatory functions. In particularly, eosinophils suppress T cell proliferation by using the protein galectin-10^7^. A common theory as to why insulin-dependent diabetes occurs is that the regulatory T cells (Tregs) have impaired T cell suppressive function. A recent report demonstrated that activated Tregs were increased in patients with T1D and had impaired suppressive function^8^. Tregs as well as eosinophils use galectin-10 to inhibit T cell proliferation^7,9^. Another common feature of Tregs and eosinophils is that eosinophils also express FOXP3^10^. Moreover, galectin-10^hi^ eosinophils are present in the blood of healthy individuals and have higher suppressive capacity^7^. Interestingly, it has been demonstrated that eosinophils interact with thymocytes in the human thymus^11^, it is possible that impaired eosinophils fail already at the very first start of the autoimmunity where they allow wrongly educated thymocytes to slip past in the selection process. Interestingly, the levels of CD4+CD8+ T cells are increased in blood of patients with T1D^12^. More recently, it was demonstrated that eosinophils have a regulatory role when co-cultured with thymocytes, and a subgroup of thymic eosinophils, expressing CD38, CD16 and CD44 among other markers, was found in human thymus but was absent in blood^13^. A very interesting study indicates that eosinophils may be involved in the selection process of thymocytes^14^. Furthermore, eosinophils support the survival of long-lived plasma cells in the bone marrow^15^ as well as contribute to isotype switch of B cells^16^. All together, these reports regarding the immunoregulatory function of eosinophils have led us to hypothesize that eosinophils could play an important role in patients with T1D and need to be investigated further. Thus, we analyzed the immune cell phenotype in blood samples from patients with T1D. We designed an antibody panel that covers the major immune cell populations with focus on eosinophils and analyzed the samples using mass cytometry. Cluster analysis and multivariate methods were performed to identify differences between the immune cell populations in patients with long-standing T1D and healthy individuals. Since the T1D immunologic pathogenesis is viewed to take place consistently over long time periods, likely similar immunologic patterns exist for individuals with shorter and longer diabetes duration. Moreover, there are age-related differences in the expression of eosinophil markers^17^ which one has to consider when interpreting the results from patients with different ages and different disease duration therefore it is very important with age -matched controls. This paper may be an important start to try to reduce the major gap of knowledge when it comes to the role of eosinophils in patients with T1D as well as an overview of major immune cell populations.

## Results

### Cluster analysis reveal differences in the immune cell groups between patients with type 1 diabetes and healthy individuals

**Figure 1:**
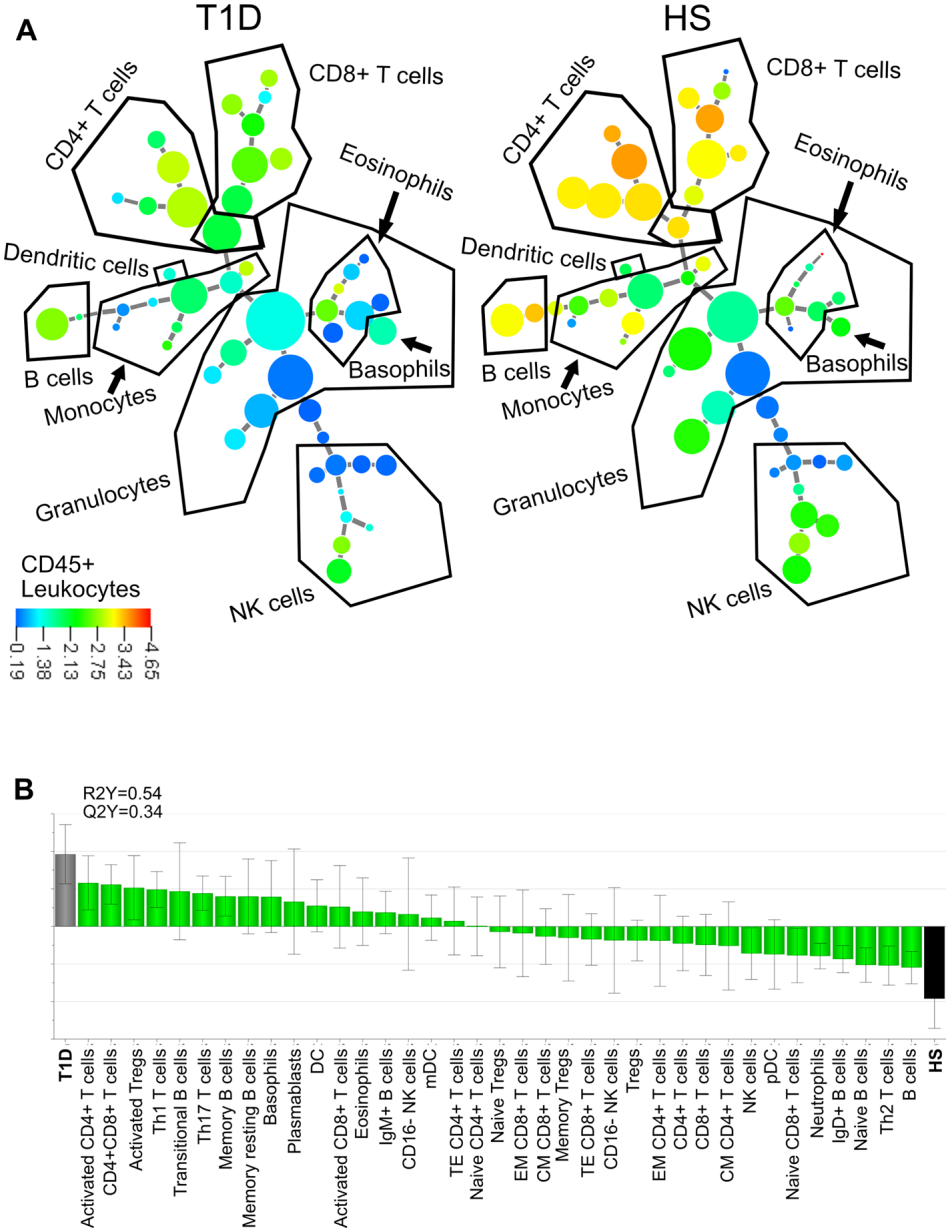
(**A**) Minimum spanning tree of major cell populations present in the blood of twelve patients with T1D and twelve healthy subjects determined by X-shift clustering analysis. The sizes of the circles represent the sizes of the populations. The color of the circles indicates the levels of CD45 expression as shown by the heat-map scale. (**B**) OPLS-DA was done to see which immune cell subsets could separate type 1 diabetes patients (T1D; n = 12) from healthy subjects (HS; n = 12). Loading plots with jackknife confidence intervals for the immune cell subsets are shown as boxes with ticks. The markers closely positioned to the patient categories are positively associated to the patient category in question. The generated two-component model had an explanatory power of 54% (a goodness of fit R2Y = 0.54) and stability of 34% (Q2Y = 0.34).

We started by making a cluster analysis covering the majority of immune cell populations by using the X-shift algorithm (Fig. 1A) in order to get a wide picture of the immune phenotype in patients with T1D. When comparing clusters between patients with T1D and healthy subjects we found differences in size and intensity of the clusters for CD4+ and CD8+ T cells, B cells, NK cells, monocytes and eosinophils (Fig. 1A). Univariate analyses of the major immune cell groups (CD4+ T cell, CD8+ T cells, B cells, NK cells, CD16+ NK cells, neutrophils, monocytes, non-classical monocytes and eosinophils) are found in Supplementary Figure 2A–I.

### Activated immune cell phenotype in patients with type 1 diabetes

To get more detailed information of all cell groups associated with T1D, multivariate analysis was used. We constructed an OPLS-DA model that could segregate samples taken from healthy subjects and from samples taken from patients with T1D based on the different cell subgroups. The model was robust with a Q2Y-value of 0.34 and with an explanatory power of 54% (R2Y = 0.54). The cell subgroups closest to the subject category are associated. We found that having T1D was strongly connected with activated CD4+ T cells, CD4+CD8+ T cells, activated Tregs, Th1 cells, transitional B cells, Th17 cells and memory B cells. In contrast, having higher levels of B cells, Th2 cells, naïve B cells and IgD+ B cells were strongly associated with healthy subjects (Fig. 1B).

### Cluster analysis of T cells and B cells discloses a higher pool of activated Tregs and memory B cells in patients with type 1 diabetes compared to healthy subjects

**Figure 2:**
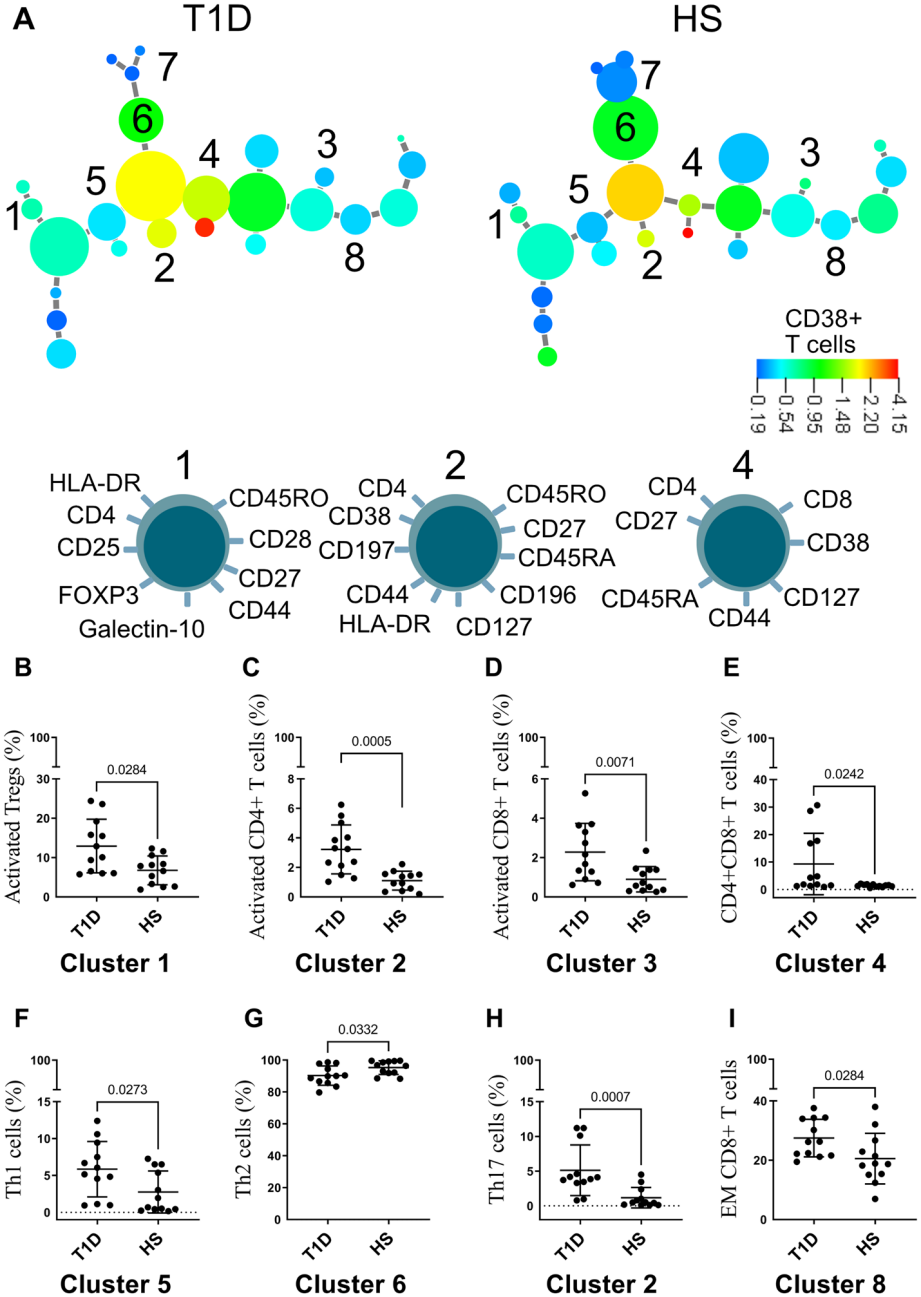
(**A**) Minimum spanning tree of T cell populations present in the blood of twelve patients with T1D and twelve healthy subjects (HS) determined by X-shift clustering analysis. The sizes of the circles represent the sizes of the populations. The color of the circles indicates the levels of CD38 expression as shown by the heat-map scale. Numbers indicate populations that are more frequent in T1D patients (1–4) or healthy donors (5–7) according to the X-shift algorithm. Phenotype of populations 1, 2 and 4 are shown below. Univariate analysis of (**B**) activated Tregs, (**C**) activated CD4+ T cells, (**D**) activated CD8+ T cells, (**E**) CD4+CD8+ T cells, (**F**) Th1 cells, (**G**) Th2 cells, (**H**) Th17 cells and (**I**) effector memory CD8+ T cells. Data are presented as mean ± SD.

To identify how the T cells and B cell subsets differed between T1D patients and healthy individuals, we continued by constructing cluster analysis for both subsets. For the analysis of T cells (Fig. 2A) we found several clusters that were larger among the T1D subjects, namely activated Tregs (cluster 1), activated CD4+ T cells (cluster 2), activated CD8+ T cells (cluster 3), CD4+CD8+ T cells (cluster 4), Th1 T cells (cluster 5), Th17 T cells that are the same cluster as the activated CD4+ T cells (cluster 2) and central memory CD8+ T cells that are the same cluster as the activated CD8+ T cells (cluster 3). In addition, there were several clusters associated with healthy subjects, namely Th2 T cells (cluster 6) and naive CD4+ T cells (cluster 7). Phenotypes of the different T cell subgroups are as follows. The activated Tregs expressed CD28, CD44, CD27, HLA-DR, galectin-10 and CD45RO (cell 1), activated Th17 cells expressed CD197, CD44, CD127, CD27 and CD38 (cell 2) and CD4+CD8+ T cells expressed CD45RA, CD27, CD44, CD127 and CD38 (cell 4). Univariate analysis confirmed that all subgroups were statistically significant, except for naïve CD4+ T cells and central memory CD8+ T cells that did not reach statistical significance, on the contraire effector memory CD8+ T cells were statistically significant (Fig. 2B–I), but this was not evident in the cluster analysis (cluster 8). In addition, we found that duration of disease correlated with activated CD4+ T cells (*P* = 0.035, r = 0.61) and inversely correlated with activated Tregs (*P* = 0.022, r = − 0.65).

**Figure 3:**
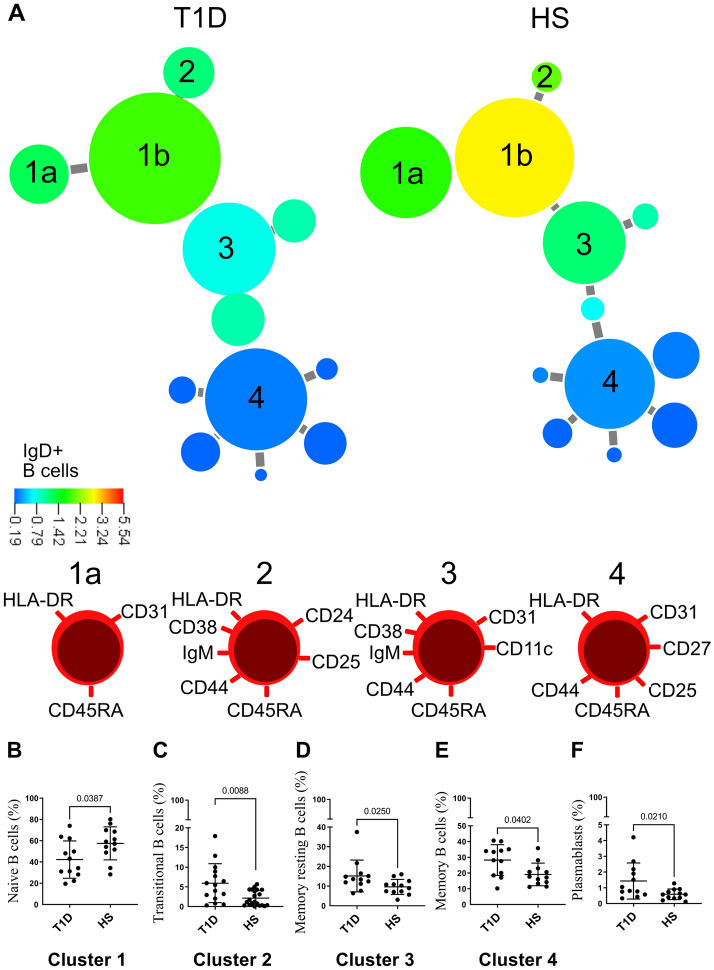
(**A**) Minimum spanning tree of B cell populations present in the blood of twelve patients with T1D and twelve healthy subjects (HS) determined by X-shift clustering analysis. The sizes of the circles represent the sizes of the populations. The color of the circles indicates the levels of IgD expression as shown by the heat-map scale. Numbers indicate populations that are more frequent in healthy donors (1a and 1b) or T1D patients (2–4) according to the X-shift algorithm. Phenotype of populations 1a, 2, 3 and 4 are shown below. Univariate analysis of (**B**) naïve B cells, (**C**) transitional B cells, (**D**) memory resting B cells, (**E**) memory B cells and (**F**) plasmablasts. Data are presented as mean ± SD.

For the analysis of B cells (Fig. 3A) we found two clusters representing the naïve B cells (cluster 1a and 1b) where cluster 1b expressing higher levels of IgD in the group of healthy subjects compared with the T1D group. Moreover, we found three clusters associated with having T1D, namely transitional B cells (cluster 2), memory resting B cells (cluster 3) and memory B cells (cluster 4). Phenotypes of the different B cell subgroups are as follows. Naïve B cells expressed HLA-DR, CD45RA and CD31 (cell 1b), transitional B cells expressed HLA-DR, CD45RA, CD38, IgM, CD44, CD25 and CD24 (cell 2), memory resting B cells expressed HLA-DR, CD45RA, CD38, IgM, CD44, CD31 and CD11c (cell 3) and memory B cells expressed HLA-DR, CD45RA, CD44, CD31, CD27 and CD25 (cell 4). Univariate analysis confirmed that all B cell subgroups were statistically significant (Fig. 3B–E). In addition, plasmablast percentage was elevated in the T1D group (Fig. 3F). Plasmablasts are not included in the cluster analysis, since it only covers all B cells expressing CD20.

### Alternate levels of eosinophil markers in patients with type 1 diabetes

**Figure 4:**
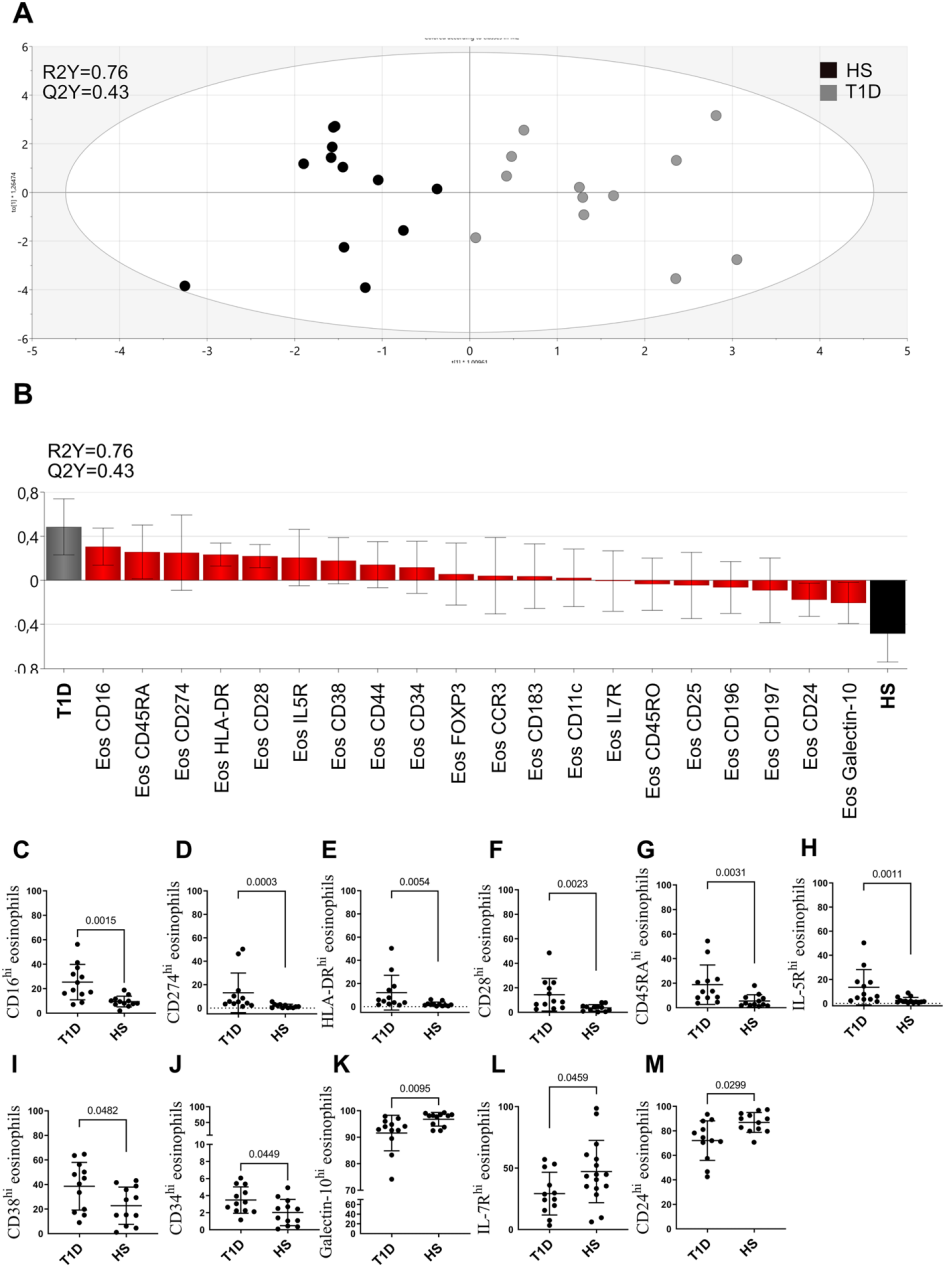
(**A**) Multivariate analysis of blood eosinophil marker levels in the study groups. “Orthogonal partial least squares-discriminant analysis” (OPLS-DA) was done to see if the eosinophilic markers (X-variables) could segregate the two study groups (Y-variables, e.g., type 1 diabetes patients (T1D; n = 12) and healthy subjects (HS; n = 12)). The generated two-component model had an explanatory power of 76% (a goodness of fit R2Y = 0.76) and stability of 43% (Q2Y = 0.43). (**B**) OPLS-DA was done to see which eosinophil markers could separate type 1 diabetes patients (T1D; n = 12) from healthy subjects (HS; n = 12). Loading plots with jackknife confidence intervals for the eosinophil markers are shown as boxes with ticks. The markers closely positioned to the patient categories are positively associated to the patient category in question. Univariate analysis of (**C**) CD16^hi^ eosinophils, (**D**) CD274^hi^ eosinophils, (**E**) HLA-DR^hi^ eosinophils (**F**) CD28^hi^ eosinophils, (**G**) CD45RA^hi^ eosinophils, (**H**) IL-5R^hi^ eosinophils, (**I**) CD38^hi^ eosinophils, (**J**) CD34^hi^ eosinophils, (**K**) galectin-10^hi^ eosinophils (**L**) IL17R^hi^ eosinophil and (**M**) CD24^hi^ eosinophil. Data are presented as mean ± SD.

Next, we examined if it was possible to differentiate patients with T1D from healthy age matched controls based on eosinophil markers using multivariate analysis. Indeed, a model capable of separating the two groups was generated (Fig. 4A) with a robustness of 43% (Q2Y = 0.43) and with an explanatory power of 76% (R2Y = 0.76). The most discriminatory parameters are shown in Fig. 4B. On the right are the parameters associated with healthy subjects and to the left are the parameters associated with T1D patients. Healthy subjects had higher levels of galectin-10, CD24, CD197, CD196, CD25 and CD45RO and patients with type 1 diabetes had higher levels of CD16, CD45RA, CD274, HLA-DR, CD28, Interleukin (IL)-5R, and CD38 (Fig. 4B). Next, univariate analysis confirmed that CD16, CD274, HLA-DR, CD28, CD45RA, IL-5R, CD38 and CD34 were elevated in patients with type 1 diabetes, whereas galectin-10, CD24 and IL-7R were lower in patients with type 1 diabetes (Fig. 4C–M).

### Cluster analysis exposes lower levels of galectin-10+ eosinophils and higher levels of immature eosinophils in patients with type 1 diabetes

**Figure 5:**
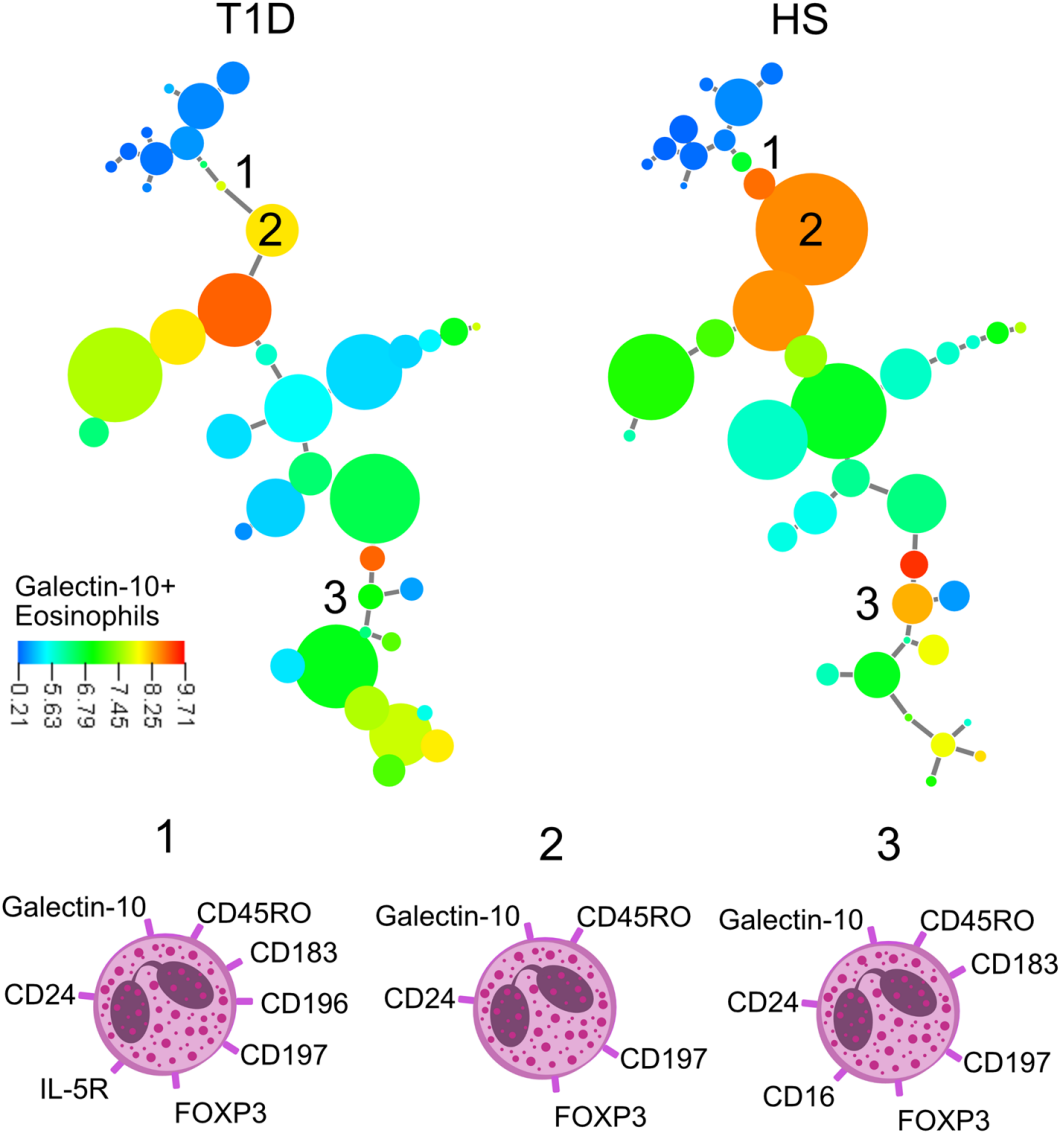
Minimum spanning tree of eosinophil populations present in the blood of twelve patients with T1D and twelve healthy subjects (HS) determined by X-shift clustering analysis. The sizes of the circles represent the sizes of the populations. The color of the circles indicates the levels of galectin-10 expression as shown by the heat-map scale. Numbers indicate populations that are more frequent in healthy donors compared with T1D patients (1, 2, 3) according to the X-shift algorithm. Phenotype of populations 1–3 are shown below.

**Figure 6:**
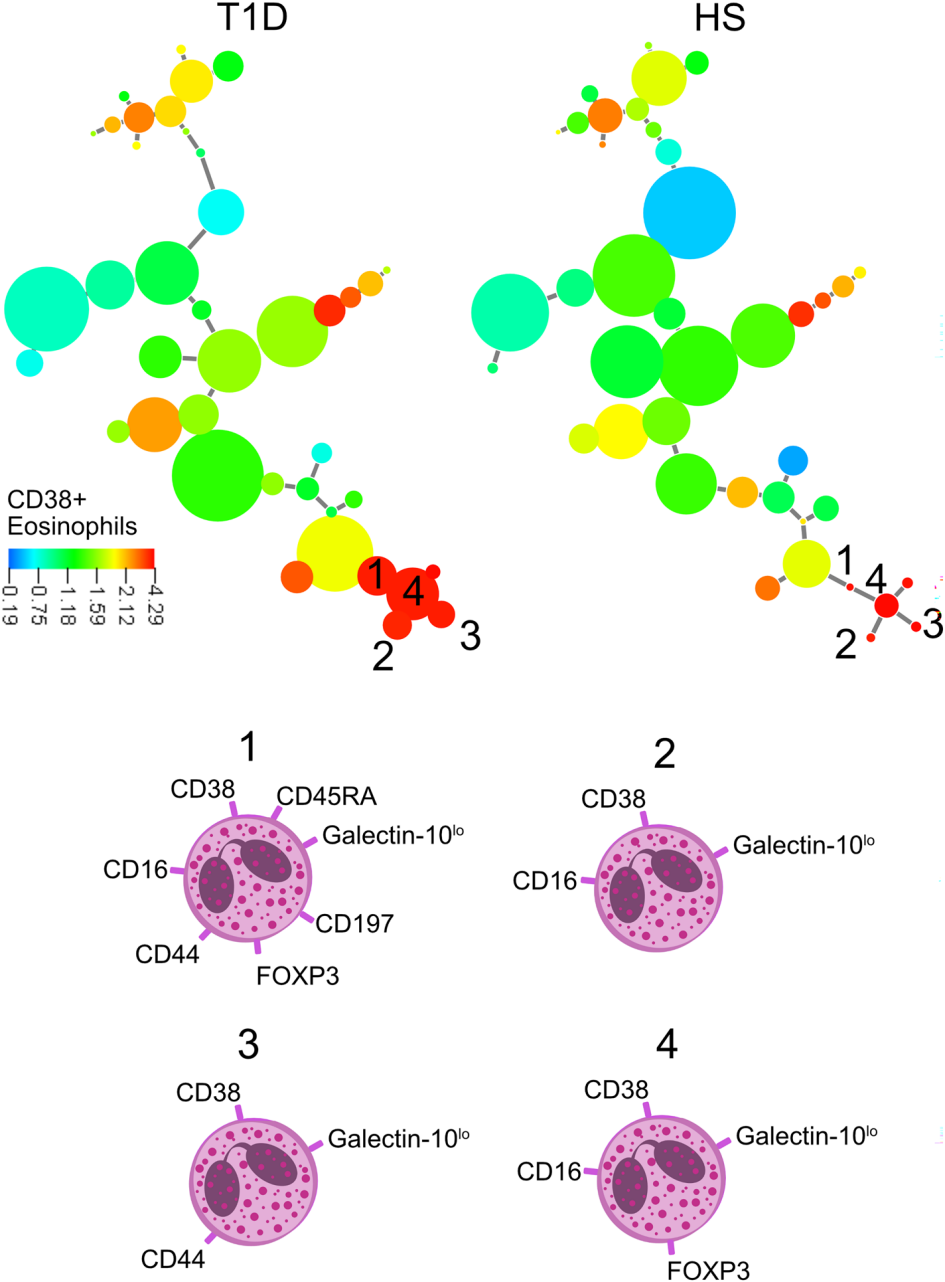
Minimum spanning tree of eosinophil populations present in the blood of twelve patients with T1D and twelve healthy subjects (HS) determined by X-shift clustering analysis. The sizes of the circles represent the sizes of the populations. The color of the circles indicates the levels of CD38 expression as shown by the heat-map scale. Numbers indicate populations that are more frequent in T1D patients compared with healthy donors (1, 2, 3, 4) according to the X-shift algorithm. Phenotype of populations 1–4 are shown below.

Next, we used cluster analysis to get a more detailed picture of different eosinophil subgroups when comparing patients with T1D to healthy subjects. The X-shift algorithm constructed 40 clusters in which healthy subjects had a bigger population of eosinophils that expressed galectin-10, than T1D patients (Fig. 5, cluster 1–3). Phenotypes of the different eosinophil subgroups are shown in Fig. 5 (cell 1–3), the most common markers for these three subgroups were galectin-10, CD24, CD45RO, FOXP3 and CD197. Cluster 1–3 consists together of 4% of the total number of eosinophils in the T1D group and 32% in the group of healthy subjects. Interestingly, cluster 1, that express IL-5R and CD196 is entirely absent in all T1D patients. On the contraire, the clusters expressing high levels of CD38, were larger for patients with T1D (Fig. 6) and common for clusters 1–4 were CD38, CD16 and galectin-10^lo^. Cluster 1–4 consists together of 7% of the total number of eosinophils in the T1D group and 0.8% in the group of healthy subjects and cluster 1 is entirely absent in the group of healthy subjects. The phenotype of these subgroups is indicative of more immature cells.

## Discussion

In this study we have examined the major immune cell groups, included in the innate and adaptive immune response, in patients with T1D. We could clearly see that several subgroups differed between T1D patients and healthy individuals. In particularly, T cells, B cells and eosinophils. In addition, the level of CD45 was lower in almost all cell groups. Interestingly, it has been shown that the gene for CD45 is downregulated in children with high risk of type 1 diabetes, while diabetic children had levels close to healthy controls^18^. The authors speculated that the low expression of CD45 could be due to impaired regulation of immune responses during the pre-diabetic period, resulting in sustained inflammation and autoimmunity^18^. There are other reports regarding CD4+ and CD8+ T cells in patients with T1D^12,19^ showing both similar and different findings compared to ours. An important difference to highlight is that many studies analyze frozen PBMC whereas we have analyzed whole blood. Although, in line with our results, Buschard et al. demonstrated an increase of activated CD4+ and CD8+ T cells in newly diagnosed patients with T1D. However, 7 months after diagnosis the increase was predominantly in CD8+ T cells^19^. Contradictory, we found that activated CD4+ T cells correlated with disease duration. Teniente-Serra et al. found a statistically significant decrease of late effector memory CD8+ T cells and a trend towards decreased levels of early effector memory CD8+ T cells in adult onset T1D patients^12^. On contraire, we found that effector memory CD8+ T cells were elevated in patients with long-standing T1D. Perhaps, this finding is more related to the time afflicted with T1D, although we did not find a correlation with disease duration in our study. However, attempts have been made to try and preserve beta cell function by depletion of effector memory T cells^20^. The increase of effector memory T cells could be a result of low levels of galectin-10^hi^ eosinophils in patients with T1D, as our cluster analysis revealed in this study. The fact that patients with T1D had higher levels of activated Tregs and that the levels of activated Tregs correlated inversely with disease duration is interesting, as there are contradictory reports whether activated Tregs are upregulated or downregulated in patients with T1D^8,12,21^. However, activated memory Tregs in patients with slow progressive T1D are functionally impaired when suppressing T cells^8^. Eosinophils are capable of suppressing all T cells alike^10,22^ and the suppression is mainly dependent of galectin-10^7^. Tregs also need galectin-10 in order to suppress T cells^9^. Suppressive eosinophils that are more potent in suppressing effector T cells than conventional eosinophils express higher levels of galectin-10^7^. One could speculate that the suppressive capacity of Tregs and eosinophils is impaired in patients with T1D perhaps due to failure of galectin-10 transport. Cappellari et al. showed that CD49d+ cells, most of which were eosinophils, was downregulated in diabetic mice which was independent of glucose levels, and they speculate that the lower levels are part of the disturbed myelopoiesis associated with diabetes. They then analyzed the gene expression of haematopoietic colonies generated before and after CD34+ stem cell mobilization and they found that patients with diabetes expressed lower levels of the PRG2 gene which encodes the eosinophil-specific marker major basic protein^4^. Suppressive eosinophils express lower levels of CD49d compared to conventional eosinophils^23^, perhaps the decreased levels of CD49d in patients with diabetes is an attempt to suppress activated T cells.

We also found that T1D patients had higher levels of CD4+CD8+ T cells, which has been shown before by Teniente-Serra et al.^12^. They showed that the CD4+CD8+ T cells that was elevated in the T1D patients were both CD45RA+CCR7+CD27+ and CD45RA-CCR7-CD27-. This is in line with our results, our cluster analysis revealed that the CD4+CD8+ T cells expressed CD27, CD38 and CD45RA, suggesting that they are immature cells. This could indicate that the selection process in thymus is somewhat impaired. Thymic eosinophils, expressing CD38, CD16 and CD44 among other markers, have been found in human thymus but are absent in blood^13^. In that report, 5% of the thymic eosinophils had that unique phenotype^13^, which is about the same level as we found in the circulation of T1D patients. The fact that we also found eosinophils with a thymic phenotype expressing CD38, CD44 and CD16 in the blood of T1D patients, which was almost absent in healthy subjects, is an additional indication that the thymus could be dysregulated in patients with T1D. Although many immune cells express CD38, eosinophils generally do not, except for the eosinophil progenitors in the bone marrow^24^. Thymic eosinophils interact with thymocytes and the majority are located in the corticomedullary junctions, where the selection process takes place^11^. Both thymic B cells and thymocytes increases in the thymus during T1D development. Diabetic mice had a significant accumulation of thymic B cells before onset of T1D^25^, and migration-related molecules that affects the T cell development in non-obese diabetic mice is altered^26^. These studies together with our results points toward that more research regarding the thymus in patients with T1D is of interest.

The activated CD4+ T cells in our cluster analysis had a Th17 cell phenotype expressing CD196. Ongoing studies to try and slow down the beta cell destruction are made by administrating monoclonal antibodies targeting IL-17 or Th17 cells, already used for patients with psoriasis^27^. The fact that these cells expressed both CD45RA and CD45RO is indeed interesting, studies have shown that newly diagnosed patients with T1D have increased numbers of lymphocytes co-expressing the CD45RA and CD45RO, that responds to stimulation with insulin and beta-cell membrane^28^. Reports have shown that eosinophils regulate Th17 cells and inversely correlate with IL-17 production in mice^29^. Thymic eosinophils express Indoleamine 2,3-Dioxygenase- (IDO), which is associated with selective apoptosis of thymocytes and Th1 cells but not of Th2 cells in mice^14^. If the regulatory functions of eosinophils would be impaired an unbalance between Th1, Th2 and Th17 could occur.

Regarding the B cells, patients with T1D had lower levels of naïve B cells and higher levels of transitional B cells, memory B cells as well as resting memory B cells, which is in line with several other reports. Ling et al. found that naïve B cells were lower in new-onset diabetic patients but this was not as evident in long-term diabetic patients compared to healthy controls^30^. Habib et al. showed that transitional B cells were increased in T1D patients and on the contraire Teniente et al. showed a decrease of transitional B cells. An important difference between the two studies is that Teniente et al. had adult-onset patients in their study whereas Habib et al. did not. Opposite to our findings Habib et al. demonstrated a decrease of memory B cells among T1D patients^31^. On the other hand, Teniente et al. showed that IgM+ memory B cells were elevated in T1D patients^12^ and Ling et al. found a trend towards increased switched memory B cells as well as higher levels of plasmablasts in patients with T1D^30^. Accordingly, we also found increased levels of plasmablasts in the T1D group. Interestingly, eosinophil-deficient mice have a significant increase in plasma cells compared to wild-type mice^32^. It has been published, that increased levels of plasmablasts possibly could enhance T cell-mediated beta cell destruction and promote the development of type 1 diabetes^30^. We speculate that altered B cell subsets are an indirect consequence of impaired eosinophils failing at suppressing the activated T cells.

In healthy individuals suppressive eosinophils express CD16^7^. We speculate that eosinophils in patients with T1D are upregulating CD16 in order to regulate activated T cells, but since the eosinophils do not express the suppressive protein galectin-10 and the adhesion molecule CD24 to the same extent as healthy subjects the suppression of and interaction with T cells is much weaker. In addition, we found that there was fewer IL-7R^hi^ eosinophils in patients with T1D. IL-7R is required for eosinophil development and survival^33^. Furthermore, it has been shown that the IL-5 levels are upregulated in plasma from patients with T1D^34^. Interestingly, one of the clusters with galectin-10^hi^ expressing eosinophils, that was entirely absent in the T1D group, also expressed IL-5R and CD196 also known as CCR6. CCR6 is the only receptor for CCL20, which is elevated in patients with T1D^35^. Perhaps higher levels of IL-5 and CCL20 in individuals with T1D, are an attempt to recruit galectin-10^hi^ expressing eosinophils which subsequently fails due to their absence in the T1D individuals. The fact that eotaxin, the eosinophil-recruiting chemokine, is up-regulated in the pancreatic lymph nodes of diabetic rats^3^ also indicates that the immune system is in need of recruiting eosinophils in patients with T1D. Common for all three clusters with galectin-10^hi^ eosinophils was the expression of CD197, also called CCR7. CCR7 is the chemokine receptor for CCL19 and CCL21, which attracts leukocytes to migrate toward the lymph nodes for lymphocyte interaction and antigen presentation.

The immune profile of the eosinophils in patients with T1D indicates that their eosinophils are less suppressive and more immature. Our multivariate model is very robust and stable where we can see a clear separation between the groups based on eosinophil markers, indicating that eosinophils in patients with T1D have an important role to play. To our knowledge this is the first study to demonstrate this wide picture of the immune cells in patients with T1D with a focus on eosinophils. In this initial analysis of eosinophil phenotype in persons with T1D and matched controls we reveal that individuals with T1D consistently had lower levels of galectin-10hi eosinophils and even entirely lacking certain subgroups of galectin-10hi eosinophils which was not observed in the non-diabetic individuals. Eosinophil-mediated suppression of activated T cells can be reverted by administration of galectin-10 antibodies and purified recombinant galectin-10 can suppress T cell proliferation on its own^7^. A large amount of the total protein mass is composed of galectin-10 and it is required for eosinophil differentiation and granulogenesis^36^, which could explain the more immature phenotype of eosinophils in patients with T1D. We believe our study can be an initial step toward unraveling the role of the eosinophils and galectin-10 in patients with T1D and we speculate that galectin-10-based treatment could be used to slow down or even hinder the destruction of insulin producing beta cells. A limitation in this study is the small sample set and therefor the current findings should be viewed as exploratory and need to be confirmed in an independent sample. It would also be of interest to investigate these findings in persons with short diabetes duration and other groups of diabetes patients. Noteworthy, we only found activated Tregs and activated CD4+ T cells to be correlated with disease duration indicating that the findings in this study are not related to time afflicted with T1D but an actual physiological difference between patients with T1D and healthy subjects. The T1D onset is generally several years before clinical onset^37^. The beta-cell destruction continues consistently over long time periods and up to 30 years after diabetes onset small amounts of endogenous insulin production can still be measured in many individuals^38^. The clinical onset is rather a time point when the pancreatic beta-cells are not sufficient to control the glucose level, than due to a sudden disturbance in immune system^39^. Hence, likely similar immunologic processes destroying the beta-cells persists over long time periods. The current findings, despite the relatively small sample, show significant differences in several different biomarkers is of great interest.

## Materials and methods

### Study design

Twelve adult patients with long-standing type 1 diabetes were recruited at NÄL Medical Hospital and Sahlgrenska University Hospital. Exclusion criteria was ongoing corticosteroid treatment. The mean age of the study participants (n = 12) was 37 years (range 24–55) and 58% were women. Age- and sex- matched controls (n = 12, age 37 years, range 23–55, 58% women) were also included for comparison. Supplementary Table 1 summarizes the patient characteristics. The study was approved by the Regional Ethical Review Board of Gothenburg, Sweden (Dnr 2020–00,668). Written informed consent was acquired from all study participants. The study protocol conformed to the ethical guidelines of the 1975 Declaration of Helsinki.

### Mass cytometry

Fresh heparinized venous blood samples were prepared for cytometry by time-of-flight (CyTOF) analysis within 24 h of donation from study participants. Erythrocytes were lyzed from the blood samples by ammonium chloride lysis (15 min, RT), and the remaining leukocytes were washed with Maxpar PBS (Fluidigm, South San Fransisco, CA). Cell suspensions were incubated with Cell ID Cisplatin (5 µM, Fluidigm, 5 min, RT) for dead cell exclusion, washed, and incubated with Fc receptor block Human TruStain FcX (BioLegend, San Diego, CA) and an antibody cocktail of surface markers (Supplementary Table 2) for 30 min, RT. The cells were washed, fixed in 1.6% formaldehyde solution (10 min RT), and permeabilized using Foxp3/Transcription Factor Staining buffer (eBioscience, San Diego, CA) for 1 h, RT. The cells were washed and incubated with antibodies for intracellular markers for 1.5 h, RT (Supplementary Table 2). After wash, the cells were incubated with 62.5 nM intercalation solution (Cell-ID DNA Intercalator-Ir [125 µM], Maxpar Fix and Perm Buffer, Fluidigm) for 45 min, RT. Next, the samples were resuspended in Maxpar PBS and stored overnight at 4 °C. Prior to sample analysis, the cells were resuspended and diluted in MilliQ H_2_O to 1 × 10^6^ cells/ml and 0.1X EQ Four Element Calibration Beads (Fluidigm) were added. Analyses were performed using a Helios CyTOF instrument with CyTOF Software v7.0. (Fluidigm) and samples were gated using FlowJo 10.8.0 software (Tree Star Inc., Ashland, OR) (Supplementary Figure 1). Data are presented as percentage of cells expressing the different markers.

### High-dimensional data analysis and statistics

CyTOF data were analyzed using X-shift clustering analysis. All cells were analyzed and mapped based on 44 channels, CD3+ T cells based on 34 channels, CD20+ B cells based on 29 channels and eosinophils based on 27 channels. Pre-gated samples with the populations of interest were uploaded to the VorteX software (version 29/06/17). Data were presented using a minimum spanning tree in which the sizes of the circles represent the sizes of the populations and the color of the circles indicates the intensity expression for the chosen markers as shown by the heat-map scale, blue indicates low intensity and red indicates high intensity. The benefit with this analysis is that VorteX analyze the data objectively and is also able to study the simultaneous expression of several markers and their co-expression in each cell population, and thus identify how many different sub-populations there are for each cell group. Multivariate analyses of pattern recognition “orthogonal projections to latent structures by means of partial least squares discriminant analysis” (OPLS-DA) were performed using the SIMCA-P (version 15.0.2) statistical package (MKS Data Analytics Solutions, Malmö, Sweden). The quality of the models was evaluated by their explanatory power (R2Y) and robustness (Q2Y). Univariate analyses regarding the expression of molecular markers and cell sub-groups were performed using GraphPad PRISM 9.2.0 software (GraphPad, San Diego, CA). Differences were compared using the Mann–Whitney test and correlation between data sets was analyzed with Spearman’s rank correlation. *P *values < 0.05 were considered statistically significant.

### Ethics approval

The study was approved by the Regional Ethical Review Board of Gothenburg, Sweden (Dnr 2020-00668 2020-05-1).

### Consent to participate

All study participants provided informed consent.

## Supplementary Information

Supplementary Information.

## Data Availability

The data that support the findings of this study are available from the corresponding author upon reasonable request.
