# Supplementary material for: Investigating immune profile by CyTOF in individuals with long-standing type 1 diabetes
Source: Sci Rep. 2023 May 20;13:8171. doi: 10.1038/s41598-023-35300-7 (PMC10199897; doi:10.1038/s41598-023-35300-7)
Supplement: Supplementary file 1 — Supplementary Information. [file 41598_2023_35300_MOESM1_ESM.docx]

# Investigating immune profile by CyTOF in individuals with long-duration type 1 diabetes mellitus

Dr Helen Larsson, PhD, MD^1,2^, Dr Sofie Albinsson Högberg, PhD^3^, Dr Marcus Lind PhD, MD^4,5,6^, Dr Hardis Rabe, PhD^3,7^ & Dr Christine Lingblom, PhD^3,8,*^

1. Department of ENT, Head and Neck Surgery, NU Hospital Group, Trollhättan, Sweden
2. Department of Otorhinolaryngology, Head and Neck Surgery, Institute of Clinical Sciences, Sahlgrenska Academy, University of Gothenburg, Gothenburg, Sweden.
3. Institute of Biomedicine, Department of Infectious Diseases, The Sahlgrenska Academy, University of Gothenburg, Gothenburg, Sweden
4. Department of Medicine, NU Hospital Group, Uddevalla, Trollhättan, Sweden.
5. Department of Molecular and Clinical Medicine, Institute of Medicine, University of Gothenburg, Gothenburg, Sweden.
6. Department of Medicine, Sahlgrenska University Hospital, Gothenburg, Region Västra Götaland, Sweden
7. RISE Research Institutes of Sweden, Bioscience and Materials, Gothenburg, Sweden
8. Department of Clinical Microbiology, Sahlgrenska University Hospital, Gothenburg, Region Västra Götaland, Sweden

**Email addresses**

Helen Larsson: helen.m.larsson@vgregion.se

Sofie Albinsson Högberg: sofie.albinsson@gu.se

Marcus Lind: marcus.lind@gu.se.

Hardis Rabe: hardis.rabe@gu.se

Christine Lingblom: christine.lingblom@microbio.gu.se

***Correspondence:** Christine Lingblom, Department of Infectious Diseases, Guldhedsgatan 10A, 41346, Gothenburg, Sweden, Phone: +46313424623, Fax: +4631411305, E-mail: christine.lingblom@microbio.gu.se

**Key words**: type 1 diabetes, CyTOF, T cells, B cells, eosinophils, human, cluster analysis

**Summary sentence**: Immune profile in patients with type 1 diabetes

**Supplementary figure S1.**


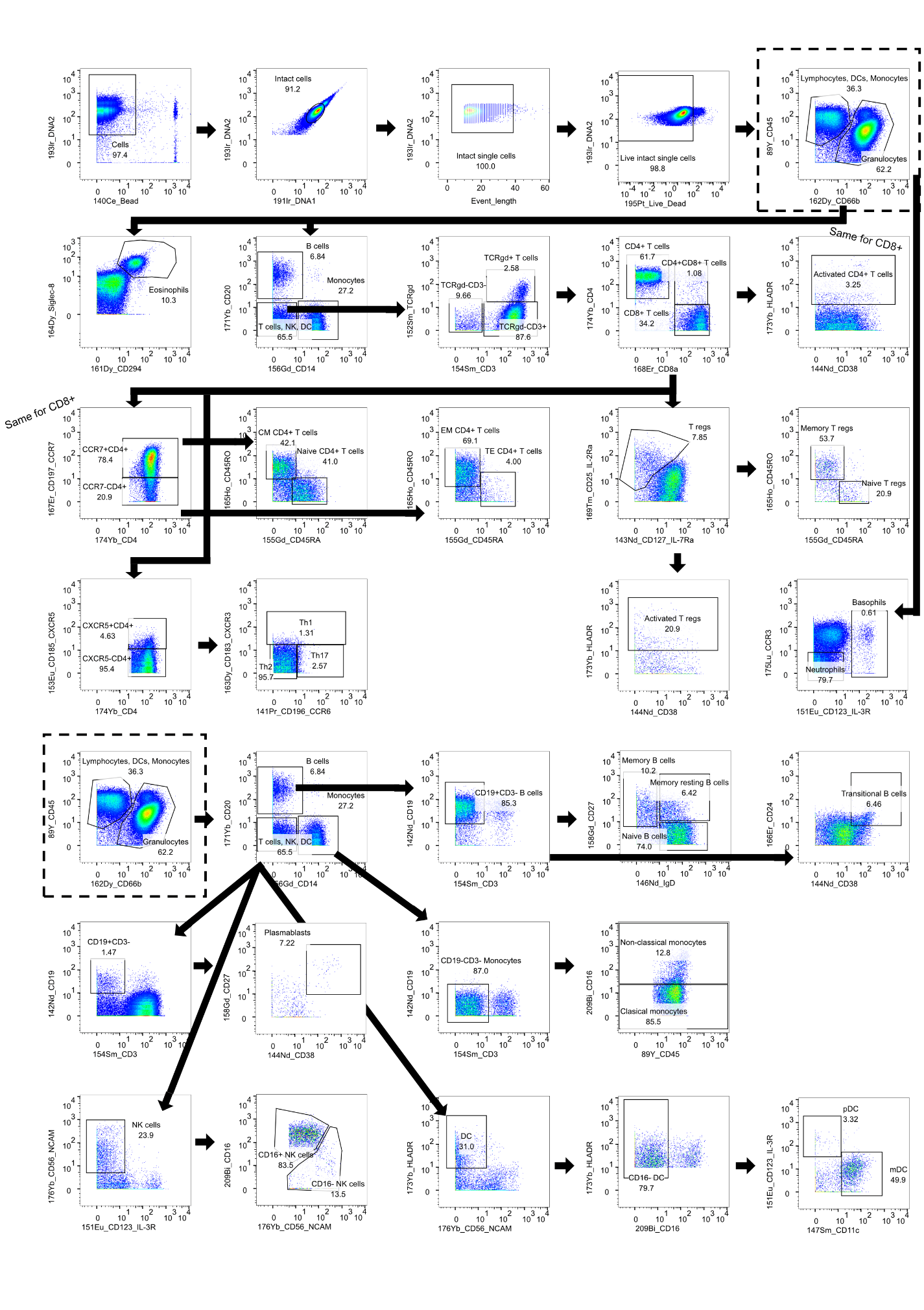


**Supplementary figure S1.** Gating strategy for samples analyzed with CyTOF. The gating is based on intact, single, live cells. Followed by gating of granulocytes from lymphocytes, dendritic cells, monocytes and NK cells. Eosinophils are gated by using CD294 and Siglec-8 from the granulocyte gate. The expression of the eosinophil markers not used in the eosinophil gating were evaluated for each sample. Gating of CD4+ T cells are depicted on the second and third row showing activated, central memory (CM), naïve, effector memory (EM), and, terminal effector (TE) CD4+ T cells, the same procedure was done for CD8+ T cells. Tregs are gated based on the expression of CD25 and CD127. Gating of Th1, Th2 and Th17 cells are shown on the fourth row and B cells on the fifth row. Rows six and seven are demonstrating the gating of plasmablast, monocytes, NK cells and dendritic cells, both myeloid (mDC) and plasmacytoid (pDC).

**Supplementary figure S2**


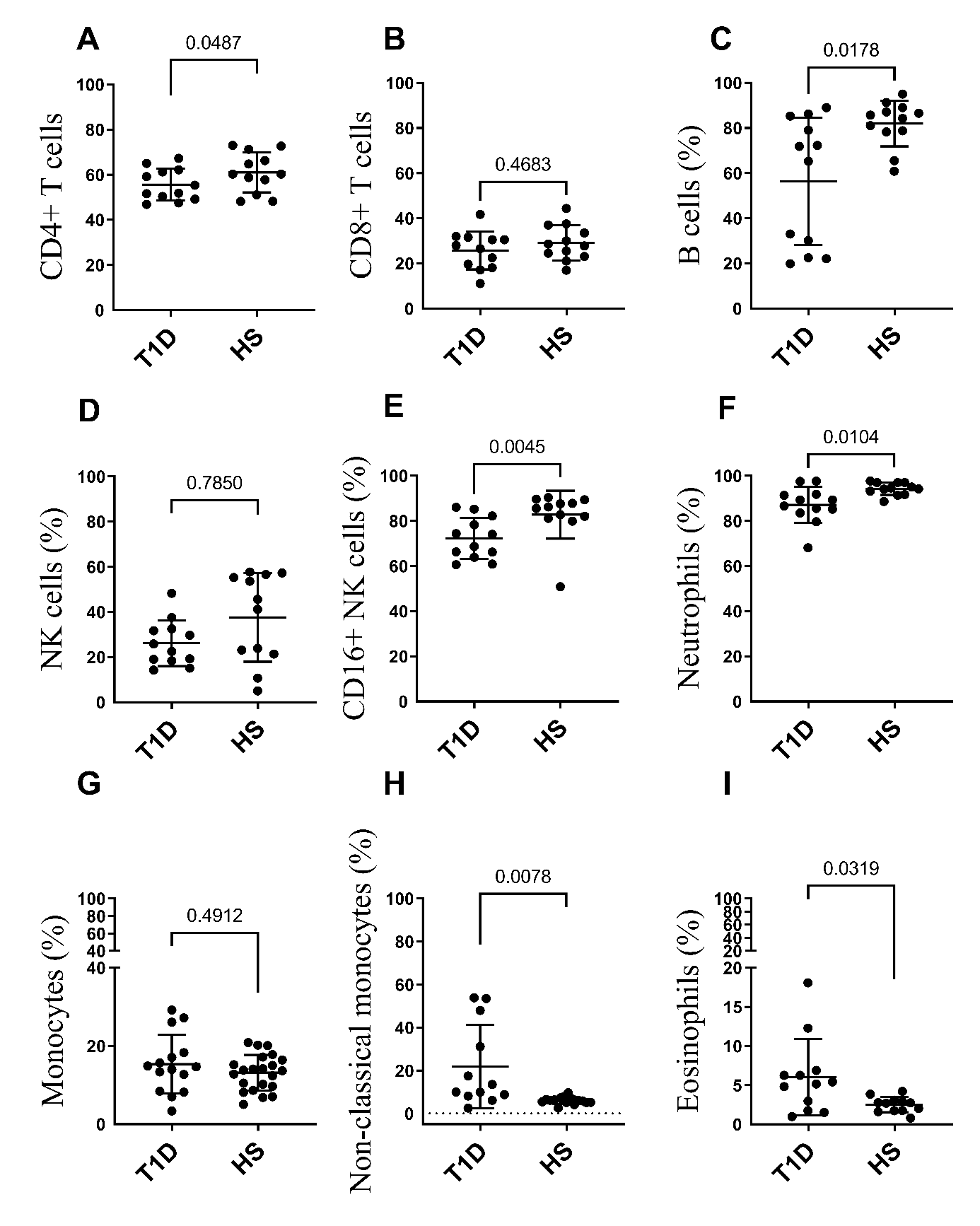
**Supplementary figure S2.** Univariate analysis of **(A)** CD4+ T cells, **(B)** CD8+ T cells, **(C)** B cells **(D)** NK cells, **(E)** CD16+ NK cells, **(F)** neutrophils, **(G)** monocytes, **(H)** non-classical monocytes and **(I)** eosinophils. Data are presented as mean ± SD.

 **Supplementary figure S3.** Correlation analysis between duration of disease and **(A)** activated CD4+ T cells and **(B)** activated Tregs

**Supplementary table S1: Demographic and clinical data of the patients with type 1 diabetes**

|  | n | % |
| --- | --- | --- |
| **Demographic data** |  |  |
| Median age (years, min/max) | 37 (24-55) | - |
| Female | 7/12 | 58 |
| **Clinical data** |  |  |
| HbA1c (mmol/mol, min/max) | 56 (44-93) | - |
| Glucose level^a^ (mmol/mol, min/max) | 11 (6-15) | - |
| Duration (years, min/max) | 24 (3-43) | - |

^a^ At the time of drawing blood

**Supplementary table S2: Antibodies used for CyTOF**

| **Antibody** | **Clone** | **Metal label** | **Source** |
| --- | --- | --- | --- |
| CD45 | HI30 | 89Y | Fluidigm |
| IgG | G18-145 | 106Cd | In house |
| RANKL | #685857 | 110Cd | In house |
| CD69 | FN50 | 111Cd | In house |
| CD31 | WM59 | 112Cd | In house |
| CD1a | HI149 | 113Cd | In house |
| CD5 | UCHT2 | 114Cd | In house |
| IgA | HP6123 | 116Cd | In house |
| CD196/CCR6 | G034E3 | 141Pr | Fluidigm |
| CD19 | HIB19 | 142Nd | Fluidigm |
| CD127/IL-7Ra | A019D5 | 143Nd | Fluidigm |
| CD38 | HIT2 | 144Nd | Fluidigm |
| IL-5R | 26815 | 145Nd | In house |
| IgD | IA6-2 | 146Nd | Fluidigm |
| CD8 | RPA-T8 | 146Nd | Fluidigm |
| CD11c | Bu15 | 147Sm | Fluidigm |
| CD274 | 29E.2A3 | 148Nd | Fluidigm |
| CD34 | 581 | 149Sm | Fluidigm |
| CD44 | IM7 | 150Nd | Fluidigm |
| CD123/IL-3R | 6H6 | 151Eu | Fluidigm |
| TCRgd | 11F2 | 152Sm | Fluidigm |
| CD185/CXCR5 | RF8B2 | 153Eu | Fluidigm |
| CD3 | UCHT1 | 154Sm | Fluidigm |
| CD45RA | HI100 | 155Gd | Fluidigm |
| CD14 | HCD14 | 156Gd | Fluidigm |
| CD27 | L128 | 158Gd | Fluidigm |
| FOXP3 | 259D/C7 | 159Tb | Fluidigm |
| CD28 | CD28.2 | 160Gd | Fluidigm |
| CD294/CRTH2 | BM16 | 161Gd | In house |
| CD66b | 80H3 | 162Dy | Fluidigm |
| CD183/CXCR3 | G025H7 | 163Dy | Fluidigm |
| Siglec-8 | 7C9 | 164Dy | Fluidigm |
| CD45RO | UCHL1 | 165Ho | Fluidigm |
| CD24 | ML5 | 166Er | Fluidigm |
| CD197/CCR7 | G043H7 | 167Er | Fluidigm |
| CD8 | SK1 | 168Er | Fluidigm |
| CD199 | L053E8 | 168Er | Fluidigm |
| CD25 | 2A3 | 169Tm | Fluidigm |
| Galectin-10 | B-F42 | 170Er | In house |
| CD20 | 2H7 | 171Yb | Fluidigm |
| IgM | MHM-88 | 172Yb | Fluidigm |
| HLA-DR | L243 | 173Yb | Fluidigm |
| CD4 | SK3 | 174Yb | Fluidigm |
| CD193/CCR3 | 5E8 | 175Lu | Fluidigm |
| CD56 | NCAM16.2 | 176Yb | Fluidigm |
| DNA1 | - | 191Ir | Fluidigm |
| DNA2 | - | 193Ir | Fluidigm |
| Cell-ID Cisplatin | - | 195Pt | Fluidigm |
| CD16 | 3G8 | 209Bi | Fluidigm |
